# MYD88 and Proinflammatory Chemokines in Aortic Atheromatosis: Exploring Novel Statin Effects

**DOI:** 10.3390/ijms24119248

**Published:** 2023-05-25

**Authors:** Konstantinos S. Mylonas, Michail Peroulis, Dimitrios Schizas, Alkistis Kapelouzou

**Affiliations:** 1 Department of Cardiac Surgery, Onassis Cardiac Surgery Center, 176 71 Athens, Greece; 2 Vascular Surgery Unit, Department of Surgery, Faculty of Medicine, University of Ioannina, 451 10 Ioannina, Greece; mperoulis@gmail.com; 3 First Department of Cardiac Surgery, Laikon General Hospital, National and Kapodistrian University of Athens, 176 71 Athens, Greece; schizasad@gmail.com; 4 Clinical, Experimental Surgery & Translational Research, Biomedical Research Foundation Academy of Athens, 176 71 Athens, Greece; akapel@bioacademy.gr

**Keywords:** MYD88, CCL4, CCL20, CCR2, atherosclerosis, statin

## Abstract

Atherosclerosis is driven by a diverse range of cellular and molecular processes. In the present study, we sought to better understand how statins mitigate proatherogenic inflammation. 48 male New Zealand rabbits were divided into eight groups, each including 6 animals. The control groups received normal chow for 90 and 120 days. Three groups underwent a hypercholesterolemic diet (HCD) for 30, 60, and 90 days. Another three groups underwent HCD for 3 months, followed by normal chow for one month, with or without rosuvastatin or fluvastatin. The cytokine and chemokine expressions were assessed in the samples of thoracic and abdominal aorta. Rosuvastatin significantly reduced MYD88, CCL4, CCL20, CCR2, TNF-α, IFN-β, IL-1b, IL-2, IL-4, IL-8, and IL-10, both in the thoracic and abdominal aorta. Fluvastatin also downregulated MYD88, CCR2, IFN-β, IFN-γ, IL-1b, IL-2, IL-4, and IL-10 in both aortic segments. Rosuvastatin curtailed the expression of CCL4, IFN-β, IL-2, IL-4, and IL-10 more effectively than fluvastatin in both types of tissue. MYD88, TNF-α, IL-1b, and IL-8 showed a stronger downregulation with rosuvastatin compared to fluvastatin only in the thoracic aorta. The CCL20 and CCR2 levels reduced more extensively with rosuvastatin treatment only in abdominal aortic tissue. In conclusion, statin therapy can halt proatherogenic inflammation in hyperlipidemic animals. Rosuvastatin may be more effective in downregulating MYD88 in atherosclerotic thoracic aortas.

## 1. Introduction

Atherosclerotic cardiovascular disease (CVD) is the primary cause of death worldwide. In 2015, more than 17 million people died from CVD, accounting for over 30% of all deaths globally [1]. Among these deaths, coronary heart disease and stroke were responsible for an estimated 7.4 million and 6.7 million, respectively. In the United States, the cost of treating CVD exceeds USD 300 billion per year, with predictions suggesting that the total cost, both direct and indirect, could reach almost USD trillion by 2030 [2].

From a pathophysiological standpoint, atherosclerosis constitutes a complex disease involving a wide array of cellular and molecular mechanisms [3,4]. Indeed, both the innate and the adaptive arms of the immune system participate in various steps of atherogenesis [5]. Within this context, toll-like receptors (TLRs) have emerged as the key players in inflammatory phenomena that drive the development of atherosclerosis [6,7]. An extensive body of literature has actually shown that a hyperlipidemic diet can induce an overexpression of proinflammatory cytokines and TLRs both in rabbit and human aortas [8,9,10]. According to recent data, highly immunogenic oxidized low-density lipoproteins (LDL) are primarily associated with the upregulation of TLR2/4 and others [11,12].

TLR signaling pathways can be categorized into two categories: a MYD88 (myeloid differentiation primary response protein 88)-dependent pathway, which triggers NF-κB (nuclear factor kappa-light-chain-enhancer of activated B cells) activation, and a TRIF (TIR domain-containing adapter-inducing IFN-β)-dependent pathway [13,14]. MYD88 is crucial for the activation of all TLRs (excluding TLR3) and some members of the IL-1 receptor family. It stimulates the MAPK (mitogen-activated protein kinase) and NF-κB signaling pathways, leading to the production and release of proatherogenic cytokines [15].

In the present animal model, we assessed the expression of MYD88 and its correlation with inflammatory markers in the setting of aortic atherosclerosis. We also explored the effect of commonly used statins on these factors.

## 2. Results

### 2.1. Effects of Atherogenesis and Statin Therapy on Aortic Cytokine mRNA Expression

The expression patterns of MYD88, NF-kB, CCL4, CCL20, CCR2, IFN-β, IFN-γ, TNF-α, IL-1b, IL-2, IL-4, IL-8, IL-10, and tIL-18 were measured against β-actin during the course of atherogenesis (G30, G60, G90, G120) and treatment with statins (GF and GR). In the control groups (C90 and C120), the mRNA expression levels of all biomarkers were very low both in the thoracic and abdominal aortas. The cytokine expression increased during the process of atherosclerosis and decreased with statin treatment. The mRNA production of all biomarkers in the thoracic aorta were higher compared to the abdominal aorta (Table 1 and Table 2).

#### 2.1.1. Thoracic Aorta

After the resumption of a normal diet (G120), MYD88, NF-kB, CCL4, IFN-β, TNF-α, IL-1b, IL-2, and IL-4 mRNA expression statistically increased compared with the G30, G60, and G90 groups in the thoracic aorta. On the other hand, G120 levels for IFN-γ, IL-8, IL-10, and IL-18 mRNA were significantly higher compared to the G30 and G60 subgroups.

Moreover, we found that the mRNA gene expression of MYD88 and CCR2 statistically decreased in the GF group compared to the G30, G60, G90, and G120 thoracic groups. In addition, NF-kB (vs. G30, G60, G90), CCL4 (vs. G30, G60, G120), CCL20 (vs. G30, G60), IFN-β (vs. G30, G120), IFN-γ (G60, G90, G120), TNF-α (vs. G30, G60, G120), IL-1b (vs. G60, G90, G120), IL-2 (vs. G90, G120), IL-4 (vs. G90, G120), IL-8 (vs. G60, G90), and IL-10 (vs. G120) were also downregulated in the thoracic GF subanalyses.

**Figure 1:**
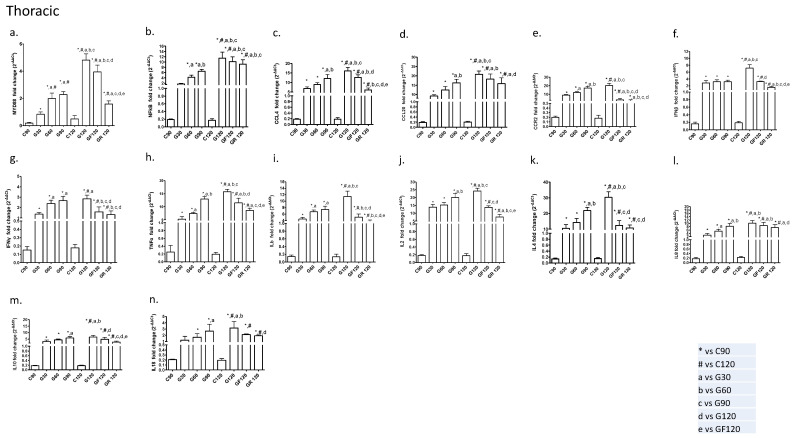
Effects of a high-fat diet and statin treatment on the thoracic rabbit aorta. (**a**) MYD88; (**b**) NF-kB; (**c**) CCL4; (**d**) CCL20; (**e**) CCR2; (**f**) IFNβ; (**g**) IFNγ; (**h**) TNFα; (**i**) IL-1b; (**j**) IL-2; (**k**) IL-4; (**l**) IL-8; (**m**) IL-10; (**n**) IL-18. Significant difference (*p* < 0.05) versus C90 (*), C120 (#), G30 (a), G60 (b), G90 (c), G120 (d), and GF120 (e).

For the first time, we found that rosuvastatin treatment is more efficient for MYD88, CCL4, IFN-β TNF- α, IL-1b, IL-2, and IL-10 downregulation in the thoracic aorta (7 out of 14), while fluvastatin treatment appeared to exert more potent effects on the rest of the biomarkers (7 out of 14) (Figure 1). In detail, the following biomarkers were significantly reduced in the GR cohorts: MYD88 (vs. G30, G90, G120, GF); NF-kB (vs. G30, G60, G90); CCL4 (vs. G60, G90, G120, GF); CCL20 (vs. G30, G120); CCR2 (vs. G30, G60, G90, G120); IFN-β (vs. G30, G60, G90, G120, GF); IFN-γ (vs. G60, G90, G120); TNF-α (vs. G30, G90, G120, GF); IL-1b (vs. G30, G60, G90, G120, GF); IL-2 (vs. G30, G60, G90, GF); IL-4 (vs. G90, G120); IL-8 (vs. G30, G120); IL-10 (vs. (G90, G120, GF); and IL-18 (vs. G120).

#### 2.1.2. Abdominal Aorta

In the G120 group of the abdominal aorta, the following cytokines were significantly upregulated: MYD88 (vs. G30, G60, G90); NF-kB (vs. G30, G60, G90); CCL4 (vs. G30, G60); CCL20 (vs. G30, G60); CCR2 (vs. G30, G60, G90); IFN-β (vs. G30, G60, G90); IFN-γ (vs. G30, G60, G90); TNF-α (vs. G30, G60, G90); IL-1b(vs. G30, G60, G90); IL-2(vs. G30, G60); IL-4 (vs. G30, G60); IL-8 (vs. G30, G60, G90); IL-10 (vs. G30, G60); and IL-18 (vs. G30, G90).

Similarly, rosuvastatin treatment (GR) significantly downregulated MYD88 (vs. G30, G60, G120), NF-kB (vs. G30, G60, G90), CCL4 (vs. G30, G90, G120), CCL20 (vs. G90, G120, GF), CCR2 (vs. G60, G90, G120, GF), IFN-β (vs. G60, G90, G120, GF), IFN-γ (vs. G30, GF), TNF-α (vs. G30, G60, G120); IL-1b (vs. G60, G90, G120), IL-2 (vs. G30, G60, G90, G120, GF), IL-4 (vs. G60, G90, G120, GF), IL-8 (vs. G30, G120), and IL-10 (vs. G90, G120, GF). As described herein, rosuvastatin treatment was more efficient for CCL20, CCR2, IFN-β, IFN-γ, IL-2, and IL-10 downregulation in the abdominal aortic segments.

**Figure 2:**
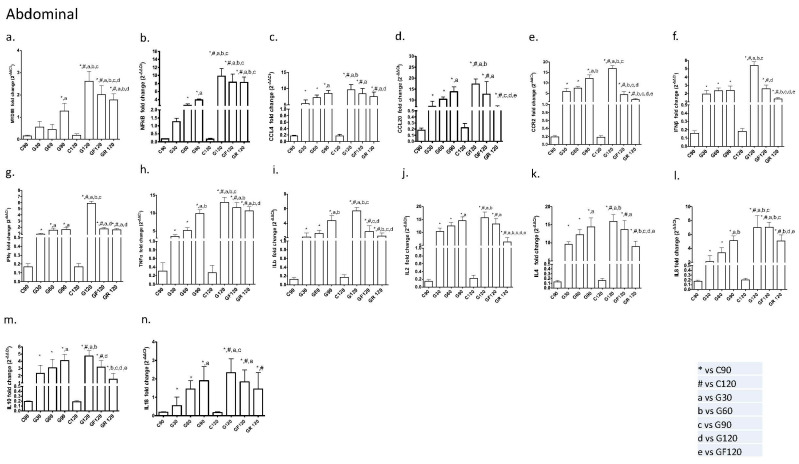
Effects of a high-fat diet and statin treatment on the abdominal rabbit aorta. (**a**) MYD88; (**b**) NF-kB; (**c**) CCL4; (**d**) CCL20; (**e**) CCR2; (**f**) IFNβ; (**g**) IFNγ; (**h**) TNFα; (**i**) IL-1b; (**j**) IL-2; (**k**) IL-4; (**l**) IL-8; (**m**) IL-10; (**n**) IL-18. Significant difference (*p* < 0.05) versus C90 (*), C120 (#), G30 (a), G60 (b), G90 (c), G120 (d), and GF120 (e).

Nevertheless, fluvastatin treatment (GF) also led to a significant attenuation in the proatherogenic biomarkers, including MYD88 (vs. G30, G60, G90, G120), NF-kB (vs. G30, G60, G90), CCL4 (vs. G30), CCL20 (vs. G30, G60), CCR2 (vs. G60, G90, G120), IFN-β (vs. G120), IFN-γ (vs. G120), TNF-α (vs. G30, G60), IL-1b (vs. G90, G120), IL-2 (vs. G30), IL-4 (vs. G30), IL-8 (vs. G30, G60, G90), IL-10 (vs. G120), and IL-18 (vs. G30). The graphical representation of these analyses can be found in Figure 2.

### 2.2. Correlations between MYD88 and Other Biomarkers during Aortic Atherogenesis and Statin Treatment

In order to assess whether MYD88-mediated signaling pathways are responsible for the progression of atherosclerosis and inflammation, we performed an analysis of the correlation coefficients between MYD88 and all other biomarkers in both aortas.

We noted a significant upregulation between MYD88 and CCL20 (G60); IFNβ (G90); NF-kB, IL18 (G120); NF-kB, CCR2, IL1b, IL18 (GF120); and CCR2 (GR120) in the thoracic aorta during the inflammatory process of atherosclerosis (Table 3). Secondly, we noticed that the MYD88 downregulation was significantly correlated with NF-kB (G90); IL4 (G120); CCL4, CCL20 (GF120); and NF-kB, IL1b (GR120) in the thoracic aorta.

We also observed that MYD88 is significantly upregulated with IL18 (G30); and TNFa and IL4 (G90) in the abdominal aorta (Table 4). Finally, we documented a significant downregulation of MYD88 with IL4 (G60); and CCL20, IL4 (GR120) in the abdominal aorta.

## 3. Discussion

Many popular cardiovascular medications seem to exhibit off-target anti-inflammatory properties [16]. Statins afford cardiovascular protection primarily by reducing LDL levels through the inhibition of HMG-CoA (3-hydroxy-3-methylglutaryl-Coenzyme A) reductase. Nevertheless, a plethora of data suggest that statins decrease cardiovascular inflammation as well. First, statins inhibit prenylated protein production and the mevalonate pathway, while inducing the Krüppel-like factor and nitric oxide synthase expression [17]. Statins also reduce endothelial cell activation and inhibit the interferon-mediated induction of major histocompatibility complex Class II expression, thereby decreasing T-cell activation [16,18]. Our study found that cytokine expression increased during the progression of atherosclerosis, but was reduced with statin treatment.

Rosuvastatin treatment significantly reduced the production of several biomarkers, including MYD88, CCL4, CCL20, CCR2, TNF-α, IFN-β, IL-1b, IL-2, IL-4, IL-8, and IL-10, in both the thoracic and abdominal aorta. Rosuvastatin reduced CCL4, IFN-β, IL-2, IL-4, and IL-10 more effectively than fluvastatin in both aortic segments. MYD88, TNF-α, IL-1b, and IL-8 showed a stronger downregulation with rosuvastatin compared to fluvastatin only in the thoracic aorta, while CCL20 and CCR2 levels reduced more extensively with rosuvastatin treatment only in abdominal aortic tissue. The IL18 production curtailed with the administration of rosuvastatin as well, but only in thoracic aortas. Fluvastatin treatment also reduced proatherogenic inflammation compared to normal diet alone, with the downregulation of MYD88, CCR2, IFN-β, IFN-γ, IL-1b, IL-2, IL-4, and IL-10 in both aortic segments. Interestingly, the CCL4 and TNF-α levels diminished with fluvastatin only in the thoracic aorta.

To explore the role of MYD88 in atherosclerosis, many groups have used MYd88−/− mice in the setting of ApoE or LDL receptor deficiency. The global deletion of MYD88 or TLR4 in these models resulted in smaller atherosclerotic plaques, at least partly due to an impaired macrophage recruitment and the lower levels of chemokines (i.e., CCL2 and CCL4) [19]. MYD88 also seems to influence macrophage function within plaques. Of note, macrophages isolated from MYD88−/− mice exhibited reduced activation, lipid accumulation, and foam cell formation in response to oxidized LDL exposure. The production of reactive oxygen species (ROS) was also reduced [20,21]. Interestingly, injecting MYd88−/− B1a cells into mice diminishes the secretion of atheroprotective IgM, which deposits in lesions and blocks T-cell infiltration and IL-1β/TNFα production to halt atherogenesis [22,23].

Work on MYD88 deletion using the Tie2-Cre driver resulted in decreased levels of IL-1β, IL-6, and CXCL1, as well as the downregulation of key adhesion molecules such as MCP1, ICAM1, and VCAM1. There are also data suggesting that the MYD88-dependent production of GM-CSF can cause monocytes to differentiate into inflammatory macrophages, thus promoting atherosclerosis [24]. The activation of MYD88-dependent pathways in aortic endothelial cells, following exposure to endotoxins (i.e., LPS and oxidized LDL), induces the expression of proprotein convertase subtilisin/kexin type 9 (PCSK9). Considering that PCSK9 is a negative regulator of the LDL receptor, these data propose an additional proatherogenic effect of MYD88 that predicates upon endothelial lipid processing [25].

Although the versatile roles of interleukins, interferons, and TNF are well established in atherogenesis, many other chemokines partake in this multi-step process. Among others, CCL20 upregulation has been documented in atherosclerotic carotid arteries and coronary plaques. Serum CCL20 levels are also increased in patients with hyperlipidemia [26]. LDL seems to induce the expression of CCL20 in VSMC, in a dose-dependent fashion through the MAPK and NF-kB-mediated pathways. The increased levels of CCL20 significantly accelerate lymphocyte and monocyte migration, whilst promoting cellular adhesion [27,28]. CCR2 appears to exert similar effects on immune cell mobilization and activation [29]. On the other hand, CCL4 triggers the release of pro-inflammatory cytokines, including IL1, IL6, and TNF-α. It also stimulates the production of ROS, and metalloproteinases 2 and 9 [30].

The current study has some limitations that need to be acknowledged. First, we included a finite number of test subjects in each group to adhere to the 3R principle (replacement, reduction, refinement) [31]. Second, our study was not designed to determine the specific mechanisms by which statins modify cytokine and chemokine production. In the future, our group will be granularly investigating the biomechanics of aortic atheromatosis, and its effects on biomarker production. Unique variations in terms of length, diameter, curvature, and branch network of the thoracic and abdominal aorta certainly affect the intrinsic hemodynamics and shear stress forces [32,33]. These variations likely account for the differences in proatherogenic cytokine expression that were observed.

## 4. Methods

### 4.1. Animal Experimental Protocol

The experimental protocol was approved by the Animal Care and Use Committee of the Athens Prefecture Veterinarian Service, Greece (K/3319/4-5-2009). All experiments took place in the animal facilities of the Center of Experimental Surgery, Biomedical Research Foundation Academy of Athens (BRFAA), according to the guidelines set by the National Research Council’s Guide for Care and Use of Laboratory Animals.

The protocol of the study has been previously published [34,35]. In this experiment, we analyzed thoracic and abdominal aortic tissue samples from 48 male New Zealand rabbits (Trompetas Breeding Laboratories; Attiki, Greece) with an average body weight of 2.8 ± 0.2 kg. The subjects were randomly divided into eight groups including six animals each, and underwent one week of acclimatization. The control groups were fed normal chow for 90 (C90) and 120 (C120) days. Three groups underwent hyperlipidemic feeds with HCD2RB19 (Mucedola, Milano, Italy) for 1 (G30), 2 (G60) and 3 (G90) months. Another three groups were fed HCD for 3 months, followed by (a) normal chow for 1 month (G120) and (b) the administration of fluvastatin (GF120) or rosuvastatin (GR120) for 1 month. Rosuvastatin (0.7 mg/kg BW) and fluvastatin (2 mg/kg BW) were administered daily per os. The rabbits were sacrificed through an intravenous overdose of sodium pentobarbital (100–120 mg/kg). The thoracic and abdominal aortas were removed and rinsed with distilled (DEPC-treated) water and shock-frozen to −140 °C for mRNA analysis.

### 4.2. mRNA Analysis of Rabbit Aortic Tissue

Total RNA was isolated from thoracic and abdominal aortas using the Trizol kit (Invitrogen, Life Technologies, New York, NY, USA), according to the manufacturer’s protocol [36]. The purity of the extracted RNA was measured with a spectrophotometer. RNA was reverse-transcribed, and the resulting cDNA was synthesized by RT (M-MLV Reverse transcriptase, Sigma), and a real-time quantitative polymerase chain reaction was performed using SYBR Green (Invitrogen, Life Technologies, NY, USA), according to our institutional protocol [37]. All experiments were repeated at least three times and the amplification signals from each target gene were normalized to b-actin (housekeeping gene). All primers for β-actin, MYD88, NF-kB, CCL4, CCL20, CCR2, IFN-β, IFN-γ, TNF-a, IL-1b, IL-2, IL-4, IL-8, IL10, and IL-18 were synthesized by TIB Molbiol (Syntheselabor GmbH, Berlin, Germany). The sequences of the primer pairs were designed with the Beacon Designer V7.0 software (Premier Biosoft International, Palo Alto, CA, USA; Table 1). The primer sequences are provided in Supplementary Table S1.

### 4.3. Statistical Analysis

All statistical calculations were performed using a one-way ANOVA for comparisons between the groups. Differences were considered significant when a two-tailed *p* < 0.05 was calculated (power analysis = 0.95, alpha = 0.05, beta = 0.05). The correlation between the measured variables was assessed through Pearson analysis. Data are presented as the mean ± standard deviation (mean ± SD). All statistical calculations were performed using GraphPad Prism version 4.03 (GraphPad Software, San Diego, CA, USA).

## 5. Conclusions

The present animal model further delineates the association between inflammatory phenomena and atherosclerosis. Several temporo-spatial differences were identified with regards to cytokine expression in the aorta. Our findings suggest that even one month of combination treatment with statins and a normal diet can halt the progression of atherosclerosis, both in the thoracic and the abdominal aorta. Furthermore, rosuvastatin may be more potent than fluvastatin in downregulating many key proatherogenic cascades, including the MYD88 pathway in the thoracic aorta. Overall, our study provides an opportunity to better understand at which stage of atherogenesis we can intervene by providing the most effective statin treatment.

## Figures and Tables

**Table 1:** Statistical analysis of the thoracic aorta mRNA expression for each biomarker across all study groups.

**Thoracic**								
**Groups**	**C90**	**G30**	**G60**	**G90**	**C120**	**G120**	**GF120**	**GR 120**
**N**	6	6	6	6	6	6	6	6
	**MYD88**							
**Mean**	0.19	0.84	1.99	2.28	0.49	4.81	3.94	1.59
**SD**	0.05	0.17	0.40	0.21	0.25	0.46	0.49	0.23
	**NF-kB**							
**Mean**	0.18	1.70	4.21	6.49	0.17	11.44	10.15	9.18
**SD**	0.02	0.21	0.81	0.74	0.04	2.25	1.80	1.63
	**CCL4**							
**Mean**	0.18	6.78	8.92	12.06	0.18	16.03	12.58	6.11
**SD**	0.03	0.92	0.93	2.05	0.05	1.80	1.42	0.99
	**CCL20**							
**Mean**	0.18	9.20	12.45	16.23	0.20	20.75	18.26	15.84
**SD**	0.03	0.65	1.80	1.87	0.03	1.81	2.68	3.06
	**CCR2**							
**Mean**	0.19	9.02	11.85	17.14	0.18	20.29	3.51	2.36
**SD**	0.02	0.74	1.17	1.76	0.06	2.60	1.55	1.09
	**IFNβ**							
**Mean**	0.16	2.87	3.19	3.21	0.18	7.16	3.25	1.55
**SD**	0.04	0.70	0.56	0.43	0.03	0.99	0.11	0.24
	**IFNγ**							
**Mean**	0.15	1.37	2.38	2.66	0.17	2.84	1.58	1.33
**SD**	0.04	0.15	0.31	0.41	0.04	0.35	0.47	0.34
	**TNFα**							
**Mean**	0.25	5.25	7.39	12.98	0.19	15.76	11.49	8.55
**SD**	0.17	1.02	0.45	0.88	0.04	0.90	1.75	0.87
	**Ilb**							
**Mean**	0.14	4.48	6.73	7.43	0.13	11.46	5.05	3.43
**SD**	0.04	0.50	0.51	0.99	0.06	1.69	1.00	0.74
	**IL-2**							
**Mean**	0.18	13.91	15.27	20.04	0.17	24.25	13.82	7.62
**SD**	0.01	1.69	1.72	2.47	0.05	1.83	0.99	1.61
	**IL-4**							
**Mean**	0.13	10.50	14.27	21.83	0.15	30.30	12.39	10.78
**SD**	0.02	2.42	2.59	2.04	0.03	3.53	3.09	1.92
	**IL-8**							
**Mean**	0.17	2.86	5.38	8.25	0.22	10.10	8.76	7.55
**SD**	0.05	1.14	1.12	1.74	0.05	1.62	1.90	1.74
	**IL-10**							
**Mean**	0.17	3.14	4.41	5.91	0.18	6.63	4.88	2.71
**SD**	0.01	1.16	0.70	0.99	0.02	1.20	1.53	0.73
	**IL-18**							
**Mean**	0.20	1.20	1.66	2.65	0.19	3.15	2.10	1.91
**SD**	0.01	0.63	0.57	1.11	0.03	1.05	0.12	0.12

Myeloid differentiation primary response 88 (MYD88); nuclear factor kappa-light-chain-enhancer of activated B cells (NF-κB); C–C chemokine ligand (CCL4); chemokine (C–C motif) ligand 20 (CCL20); C–C chemokine receptor type 2 (CCR2); interferon beta (IFN-β); interferon gamma (IFN-γ); tumor necrosis factor alpha (TNF-α); interleukin 1b (IL-1b); interleukin 2 (IL-2); interleukin 4 (IL-4); interleukin 8 (IL-8); interleukin 10 (IL-10); interleukin 18 (IL-18).

**Table 2:** Statistical analysis of the abdominal aorta mRNA expression for each biomarker across all study groups.

**Abdominal**								
**Groups**	**C90**	**G30**	**G60**	**G90**	**C120**	**G120**	**GF120**	**GR 120**
**N**	6	6	6	6	6	6	6	6
	**MYD88**							
**Mean**	0.16	0.55	0.44	1.27	0.18	2.61	2.01	1.76
**SD**	0.03	0.25	0.22	0.35	0.06	0.44	0.40	0.28
	**NF-kB**							
**Mean**	0.18	1.25	2.57	3.88	0.16	9.82	8.32	8.26
**SD**	0.02	0.21	0.32	0.25	0.05	1.89	2.05	1.35
	**CCL4**							
**Mean**	0.17	5.37	7.21	8.55	0.17	9.66	8.39	7.52
**SD**	0.02	1.09	0.77	0.86	0.05	1.63	1.60	1.42
	**CCL20**							
**Mean**	0.18	7.06	10.4	13.83	0.22	17.33	12.70	6.21
**SD**	0.03	2.48	1.00	2.24	0.07	2.32	5.72	1.15
	**CCR2**							
**Mean**	0.19	5.88	7.45	12.16	0.18	16.84	4.31	1.98
**SD**	0.02	1.56	0.91	1.57	0.04	1.36	1.53	0.6
	**IFNβ**							
**Mean**	0.16	1.96	2.36	2.40	0.18	5.37	2.61	1.35
**SD**	0.02	0.44	0.33	0.50	0.03	0.48	0.45	0.20
	**IFNγ**							
**Mean**	0.16	0.88	1.50	1.60	0.17	5.91	1.72	1.57
**SD**	0.03	0.13	0.32	0.33	0.03	0.34	0.23	0.23
	**TNFα**							
**Mean**	0.30	347	514	9.88	0.27	12.98	11.58	10.58
**SD**	0.19	0.47	0.76	1.06	0.16	1.40	1.34	1.22
	**Ilb**							
**Mean**	0.12	2.11	2.60	4.36	0.17	5.69	2.85	2.22
**SD**	0.04	0.54	0.44	0.69	0.05	0.46	0.82	0.44
	**IL-2**							
**Mean**	0.15	10.35	12.50	14.50	0.22	15.67	13.22	6.31
**SD**	0.03	1.30	1.37	1.51	0.07	2.25	2.16	1.66
	**IL-4**							
**Mean**	0.12	9.59	12.27	14.42	0.15	15.92	13.65	8.96
**SD**	0.03	0.67	1.36	2.48	0.04	1.87	2.48	1.45
	**IL-8**							
**Mean**	0.173	2.08	3.38	5.13	0.19	7.01	7.06	5.08
**SD**	0.02	0.89	0.72	0.67	0.04	1.72	0.70	0.83
	**IL-10**							
**Mean**	0.19	2.33	3.10	4.1	0.18	4.71	3.18	1.53
**SD**	0.01	1.15	1.16	0.83	0.02	0.71	0.89	0.79
	**IL-18**							
**Mean**	0.18	0.54	1.44	1.90	0.17	2.33	1.83	1.45
**SD**	0.03	0.46	0.45	0.76	0.03	0.76	0.65	0.89

Myeloid differentiation primary response 88 (MYD88); nuclear factor kappa-light-chain-enhancer of activated B cells (NF-κB); C–C chemokine ligand (CCL4); chemokine (C–C motif) ligand 20 (CCL20); C-C chemokine receptor type 2 (CCR2); interferon beta (IFN-β); interferon gamma (IFN-γ); tumor necrosis factor alpha (TNF-α); interleukin 1b (IL-1b); interleukin 2 (IL-2); interleukin 4 (IL-4); interleukin 8 (IL-8); interleukin 10 (IL-10); interleukin 18 (IL-18).

**Table 3:** Correlations between MYD88 and other biomarkers in the thoracic aorta.

	MYD88	MYD88	MYD88	MYD88	MYD88	MYD88
Group	G30	G60	G90	G120	GF120	GR120
Biomarker						
NFKB			−0.04	+0.001	+0.04	−0.09
			(−0.82)	(0.45)	(0.81)	(−0.64)
CCL4					−0.02	
					(−0.881)	
CCL20		+0.02			0.01	
		(0.86)			(0.89)	
CCR2					+0.03	+0.04
					(−0.85)	(0.82)
IFNβ			+0.01			
			(0.89)			
IFNγ						
TNFa						
ILb					+0.02	−0.01
					(0.86)	(−0.91)
IL2						
IL4				−0.02		
				(−0.86)		
IL8						
IL10						
IL18				+0.02	+0.03	
				(0.86)	(0.83)	

Correlations between MYD88 and other biomarkers in the thoracic aorta. Green boxes are statistically significant; red boxes are not statistically significant. Upregulation symbol ‘’+’’; downregulation symbol “−”. *p* < 0.005, parenthesis indicates the correlation coefficient.

**Table 4:** Correlations between MYD88 and other biomarkers in the abdominal aorta.

	MYD88	MYD88	MYD88	MYD88	MYD88	MYD88
Group	G30	G60	G90	G120	GF120	GR120
Biomarker						
NFKB						
CCL4						
CCL20						0.001
						(0.97)
CCR2						
IFNβ						
IFNγ						
TNFa						
ILb			+0.02			
			(0.50)			
IL2						
IL4		−0.008	+0.04			0.04
		(−0.92)	(0.81)			(−0.82)
IL8						
IL10						
IL18	+0.01					
	(0.89)					

Correlations between MYD88 and other biomarkers in the thoracic aorta. Green boxes are statistically significant; red boxes are not statistically significant. Upregulation symbol ‘’+’’; downregulation symbol “−”. *p* < 0.005, parenthesis indicates the correlation coefficient.

## Data Availability

Raw data may be provided by the authors upon request.

## Supplementary Materials

The supporting information can be downloaded at: https://www.mdpi.com/article/10.3390/ijms24119248/s1.
