# Supplementary material for: MYD88 and Proinflammatory Chemokines in Aortic Atheromatosis: Exploring Novel Statin Effects
Source: Int J Mol Sci. 2023 May 25;24(11):9248. doi: 10.3390/ijms24119248 (PMC10252457; doi:10.3390/ijms24119248)
Supplement: Supplementary file 1 [file ijms-24-09248-s001.zip › Supplementary Table S1.Primers.pdf]

Supplementary Table S1. Primer sequences.

Rabbit Target Gene	Forward Primer	Reverse Primer	Target Size	NM
**b actin**	CCATGTACGTGGCCATCCAG	TCTTCATGAGGTAGTCGGTCAGGTC	148nts	NM_001101683
**MYD88**	CCCTTTGTCTCTCGACTCTTGG	TACGAGAACAGCCACTGCCC	125nts	Trace Archive
**NF-κB**	ATGCCAATGCCCTCTTCGACT	CGTGACTTCCAGCAGATCCCT	122nts	Trace Archive
**CCL4**	GAGACCACCAGCCTCTGCTC	TCAGTTCAGTTCCAAGTCATCCAC	123nts	NM_001082196
**CCL20**	TATCGTGGGCTTCACACAGC	CCATTCCTTCTTCGGATCTGC	115nts	Trace Archive
**CCR2**	GGTTGCTGAGAAGCCTGACACGC	CAGGTCTGTATTCTTCAACAAGCCCTCG	125nts	Trace Archive
**IFNβ**	TCCAACTATGGCACGGAAGTCT	TTCTGGAGCTGTTGTGGTTCCT	133nts	XM_002707968
**IFNγ**	TGCCAGGACACACTAACCAGAG	TGTCACTCTCCTCTTTCCAATTCC	127nts	NM_001081991

**TNF-a**	CTGCACTTCAGGGTGATCG	CTACGTGGGCTAGAGGCTTG	133nts	NM_001082263
**IL-1b**	TTGAAGAAGAACCCGTCCTCTG	CTCATACGTGCCAGACAACACC	128nts	NM_001082201
**IL-2**	GCCCAAGAAGGTCACAGAATTG	TGCTGATTGATTCTCTGGTATTTCC	128nts	NM_001163180
**IL-4**	CGACATCATCCTACCCGAAGTC	CCTCTCTCTCGGTTGTGTTCTTG	122nts	NM_001163177
**IL-8**	CCACACCTTTCCATCCCAAAT	CTTCTGCACCCACTTTTCCTTG	122nts	NM_001082293
**IL10**	CTTTGGCAGGGTGAAGACTTTC	ACTGGATCATCTCCGACAAGG	126nts	NM_001082045
**IL-18**	ACCAAGGACAGCAACCTGTGTT	ACAGAGAGGCTTACAGCCATGC	120nts	NM_001122940

*b actin; Myeloid differentiation primary response 88 (*MYD88*); Nuclear factor kappa-light-chain-enhancer of activated B cells (NF-κB); C-C chemokine ligand (CCL4); Chemokine (C-C motif) ligand 20 (CCL20); C-C chemokine receptor type 2 (CCR2); Interferon beta (IFN-β);* Interferon gamma *(*IFN-*γ); tumor necrosis factor alpha (TNF-α); interleukin 1b (IL-1b); interleukin 2 (IL-2); interleukin 4 (IL-4); interleukin 8 (IL-8); interleukin 10 (IL-10); interleukin 18 (IL-18).*
